# Autoencoder Based Feature Selection Method for Classification of Anticancer Drug Response

**DOI:** 10.3389/fgene.2019.00233

**Published:** 2019-03-27

**Authors:** Xiaolu Xu, Hong Gu, Yang Wang, Jia Wang, Pan Qin

**Affiliations:** ^1^Faculty of Electronic Information and Electrical Engineering, Dalian University of Technology, Dalian, China; ^2^Institute of Cancer Stem Cell, Dalian Medical University, Dalian, China; ^3^Department of Breast Surgery, Institute of Breast Disease, Second Hospital of Dalian Medical University, Dalian, China

**Keywords:** anticancer drug response, autoencoder, classification model, feature selection, random forest

## Abstract

Anticancer drug responses can be varied for individual patients. This difference is mainly caused by genetic reasons, like mutations and RNA expression. Thus, these genetic features are often used to construct classification models to predict the drug response. This research focuses on the feature selection issue for the classification models. Because of the vast dimensions of the feature space for predicting drug response, the autoencoder network was first built, and a subset of inputs with the important contribution was selected. Then by using the Boruta algorithm, a further small set of features was determined for the random forest, which was used to predict drug response. Two datasets, GDSC and CCLE, were used to illustrate the efficiency of the proposed method.

## 1. Introduction

The prediction of drug responses for individual patients is an essential issue in the research of precision medicine. It is known that the drug response for various patients can be different (Wilkinson, 2005). Thus, there are different therapeutic effects when using the same anticancer drug for a cohort of patients (Dong et al., 2015). It has been suggested that the patients with similar response to an anticancer drug can have similar genetic features, like gene mutations and expressions (Wang et al., 2017). These features can be used as the biomarkers to predict the drug response (La Thangue and Kerr, 2011).

Because the clinical trials are of high time and economic costs, the researchers prefer to use the cell lines obtained from the cancer patients for investigating drug responses. These investigations lead to several drug response databases, like Genomics of Drug Sensitivity in Cancer (GDSC) (Yang et al., 2012) and Cancer Cell Line Encyclopedia (CCLE) (Barretina et al., 2012). By using these databases, constructing models for the prediction of drug response becomes feasible. Primarily, researchers always use IC50 (Barretina et al., 2012; Garnett et al., 2012), which indicates the concentration required for 50% inhibition *in vitro*, to measure the sensitivity of drug response. Taking IC50 as the dependent variable, linear regression models, including ridge regression, lasso, and elastic net, were developed to predict drug response (Barretina et al., 2012; Garnett et al., 2012; Basu et al., 2013; Iorio et al., 2016). Further complex models, like support vector regression, artificial neural network, and random forest (RF), were also constructed for this purpose (Riddick et al., 2010; Menden et al., 2013; Ammad-Ud-Din et al., 2014; Ammad-ud din et al., 2016; Costello et al., 2014; Ospina et al., 2014; Cichonska et al., 2015; Dong et al., 2015; Zhang et al., 2015). Neto et al. (2014) proposed the STREAM algorithm that combined a Bayesian inference strategy with ridge regression for the prediction of drug response. Besides the regressions, several network-based models were also proposed (Wang et al., 2014; Fey et al., 2015; Zhang et al., 2015). Model ensembles have also been considered by some works (Wan and Pal, 2014; Cortés-Ciriano et al., 2015). Meanwhile, deciding whether an individual patient is sensitive or not to the anticancer drugs is meaningful for treatment. By setting a proper threshold value for IC50, drug response can be divided into two categories: sensitivity and non-sensitivity. In this case, classification models can be fitted for predicting drug response. To this end, the recommender system, naive Bayes classifier and support vector machine have been used (Barretina et al., 2012; Dong et al., 2015; Suphavilai et al., 2018).

Nilsson et al. (2007) indicated that the appropriate selection of small feature set gives the best possible classification results. Thus, selecting an appropriate feature set from a large number of genetic feature candidates is a crucial issue for classification models for predicting drug response. In this paper, we developed a drug response prediction model, called AutoBorutaRF, by using autoencoder (Liou et al., 2008) and Boruta algorithm (Kursa et al., 2010) for feature selection and RF for classification. We first constructed the autoencoder network (Liou et al., 2008), which is a type of artificial neural network, for the reduction of genetic features. By using the Gedeon method (Gedeon, 1997), we initially reduced the total number of features. We further selected a smaller feature set feasible for RF by using the Boruta algorithm. By applying AutoBorutaRF to GDSC and CCLE, we proved that our proposed method is of excellent prediction accuracy. We further analyzed the biomarkers obtained from the lung cell lines in GDSC by the proposed feature selection method.

## 2. Materials and Methods

### 2.1. Datasets and Preprocessing

In this research, we used two datasets, including GDSC (Garnett et al., 2012) and CCLE (Barretina et al., 2012). The datasets were downloaded by using R package PharmacoGx (Smirnov et al., 2015). We used the sensitivity measure IC50 (Barretina et al., 2012; Garnett et al., 2012) as the response variable (denoted by *y*_*rs, c*_) for cell line *c*. We used three types of genetic features as the explanatory variables, including the gene expression (denoted by ***x***_*rna, g*_), the single-nucleotide mutation (denoted by ***x***_*snv, g*_), and the copy number alternation (denoted by ***x***_*cna, g*_) for gene *g*. Note that the elements in ***x***_*rna, g*_ and ***x***_*cna, g*_ are real-valued; the elements in ***x***_*snv, g*_ are binary-valued, i.e., “1” for mutation and “0” for wild type. In the two datasets, some cell lines missed the values of the response variable, the single-nucleotide mutation features, and the copy number alteration features. There was no missing value in the gene expression features. We first removed the features with the cell lines missing values more than 50%. Then, we removed the cell lines with more than 50% features missing values from the datasets. For the remaining cell lines with missing values, we used a weight mean method to compensate the missing values as follows:

1. Let $z_{c,g}^{*}$ denote the missing value for the cell line *c* in the response variable or the genetic feature *g*. Let ***x***_*rna, c*_ denote the vector of gene expression features for the cell line *c*.
2. Assume the cell line *k* has no missing data for the features involved in $z_{c,g}^{*}$. The diversity between the cell lines *c* and *k* is obtained by $d\left(c,k\right)=||{\text{x}}_{rna,c}-{\text{x}}_{rna,k}|{|}_{2}^{2}$. Search *K* cell lines nearest to *g* with respect to *d*(*c, i*).
3. If *g* is the response variable or the copy number alternation feature, $z_{c,g}^{*}$ is compensated by
  $$\begin{array}{l} {\hat{z}}_{c,g}^{*}=\overset{K}{\underset{k=1}{\sum }}\frac{d\left(c,k\right)}{\overset{K}{\underset{k=1}{\sum }}d\left(c,k\right)}z_{k,g} \end{array}$$
4. If *g* is the single-nucleotide mutation feature, *z*_*c, g*_ is compensated by
  $${\hat{z}}_{c,g}^{*}=\{\begin{array}{ll} 1 & \sum_{k=1}^{K}1(z_{k,g}=1)>\sum_{k=1}^{K}1(z_{k,g}=0)\\ 0 & \text{otherwise} \end{array}$$
  where **1**() = 1 for the true statement in the parenthesis and **1**() = 0 for the negative statement in the parenthesis.

We set *K* = 10 for the preprocessing of GDSC and CCLE datasets.

### 2.2. Label Assignment for Cell Lines According to IC50

This research is to construct classification models for predicting how the cell lines respond to the drugs under study. The drug responses can be divided into two categories: “sensitivity" and “non-sensitivity” (Liu et al., 2016). So far, several works have used various threshold values of IC50 to classify the drug responses (Brubaker et al., 2014; Li et al., 2015). Brubaker et al. (2014) used a hard threshold 0.1 to label sensitivity for IC50< 0.1 and to label non-sensitivity (i.e., resistance in this work) for IC50≥ 0.1. However, by investigating the histograms of IC50, we found that the statistics of drugs are various. It can be supposed that the decision of labels should be driven by the data of individual drugs. To this end, we adopted the strategy introduced in Li et al. (2015), which used the median of the observed IC50 values as a data-driven threshold. We labeled a cell line as “sensitivity” if its IC50 is smaller than the median overall the cell lines for an individual drug. We labeled a cell line “non-sensitivity” if its IC50 is equal to or larger than the median overall the cell lines for an individual drug.

### 2.3. Classification Model and Feature Selection for Predicting Drug Response

#### 2.3.1. Classification Model

The drug response data are often of imbalanced classifications. Because RF is outstanding for the imbalanced classification problem, we used it as the classification model. In RF, we used classification and regression trees (CART) algorithm as the basic classifier. RF randomly generalizes 1,000 CARTs. Each CART is trained by using ⌈0.632 × *N*_*sample*_⌉ bootstrapping samples, where *N*_*sample*_ is a total of cell lines. The ultimate results were determined through voting with the prediction results of all CARTs.

#### 2.3.2. Feature Selection With the Autoencoder and Boruta Algorithm

**Figure 1:**
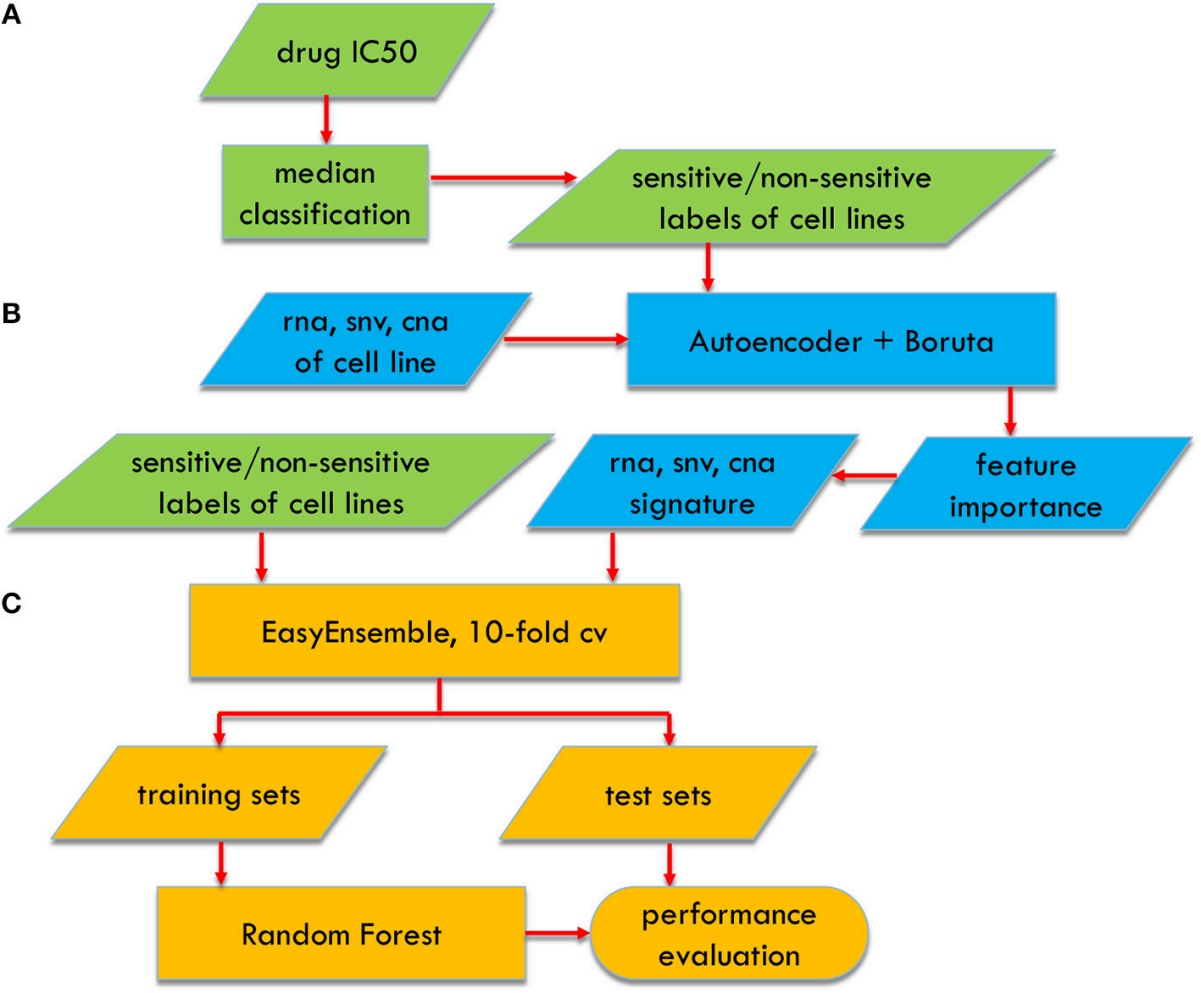
Flowchart of AutoBorutaRF for predicting anticancer drug response, which includes three parts: **(A)** data preprocessing, **(B)** feature selection, and **(C)** classifier constructing.

Feature selection is crucial for improving the prediction performance of the classification models. We used the Boruta algorithm, which aims to the feature selection problem for RF (Kursa et al., 2010) (Figure 1). The considerable cardinality of the feature candidate set leads to the curse of dimensionality for the Boruta algorithm. Thus, we first used the autoencoder network, to roughly screen out the features to a proper dimension. The detailed two-stepwise feature selection procedure is described as follows:

Step 1: We trained two single-hidden-layer autoencoder networks, with hyperbolic tangent being the activation functions, for screening out the features of the gene expression and the features of the copy number alteration, respectively. Different from the straight application of the hidden layers of the autoencoder, we used Gedeon method (Gedeon, 1997) to calculate the proportional contributions to select the significant genes. The contribution of the *i*th input (gene) to the *j*th output (gene) is calculated as
  $$\begin{array}{l} Q_{ij}=\overset{K}{\underset{k=1}{\sum }}\left(P_{ik}\times P_{kj}\right) \end{array}$$
  Here *K* denotes the total number of the neurons of the hidden layer. *P*_*ik*_ is the contribution of the *i*th input to the *k*th neuron of the hidden layer calculated by
  $$\begin{array}{l} P_{ik}=\frac{\left|W_{ik}\right|}{\overset{G}{\underset{i^{*}=1}{\sum }}|W_{i^{*}k}|} \end{array}$$
  with *G* being the total number of the inputs and $W_{i^{*}k}$s being the weights linking the corresponding neuron couples. *P*_*kj*_ is the contribution of the *k*th neuron of the hidden layer to the *j*th output, whose calculation is similar to that of *P*_*ik*_. The total contribution of the *i*th input is calculated by
  $$\begin{array}{l} q_{i}=\overset{G}{\underset{j=1}{\sum }}\frac{Q_{ij}}{\overset{G}{\underset{i^{*}=1}{\sum }}Q_{i^{*}j}} \end{array}$$
  We ranked the inputs of the autoencoder in the descending order with respect to *q*_*i*_ and removed the last 50% features. We also removed the features, whose means of correlation coefficients with other features were more than 0.95.
Step 2: From the features obtained by Step 2, the Boruta algorithm was used to select features for RF as follows:
  2-1. Extend the dataset by adding copies of all the features obtained by Step 1.
  2-2. Shuffle the values of the copied features, called shadow features, to remove their correlations with the response variable, i.e., IC50.
  2-3. The shadow features are combined with the original ones.
  2-4. Run a random forest classifier on the combined dataset and perform a variable importance measure, in which the mean decrease accuracy (MDA) is used.
  2-5. Z score is calculated by dividing MDA with the standard deviation of accuracy loss.
  2-6. Find the maximum Z score among shadow attributes (MZSA).
  2-7. The features with importance significantly lower than MZSA are permanently removed from the dataset. The features with importance significantly higher than MZSA are retained as important features.
  2-8. The shadow features are removed from the dataset.
  2-9. Repeat the above steps until for the prefixed iterations (200 was prefixed in our study), or all the retained features are important features.

### 2.4. EasyEnsemble for Imbalanced Datasets

The total number of cell lines sensitive to drugs is much smaller than that of cell lines non-sensitive to drugs. Thus, the datasets in this research are the class imbalance. Let N and R denote the sample set of majority class (non-sensitivity) and that of minority class (sensitivity), respectively. The imbalance ratio IR=|N|/|R| is used to measure the class imbalance, with | · | being the cardinality of a set. For the various drugs under study, the values of IR are different. In this research, for the drugs with IR≤ 2, the feature selection and classification method were directly used; for the drugs with IR >2, we used EasyEnsemble (Liu et al., 2009) resampling strategy to deal with the imbalance class problem. The core procedure of EasyEnsemble used here is described as follows:

1. Equally divide N into *T* subsets $\left\{N_{i}|i=1,2,\cdots \;,T\right\}$, with *T* = ⌊IR⌋. Such that $\left|N_{i}|\approx |R\right|$.
2. The RF classifier *F*_*i*_(*x*) is constructed on each training subsets $\left\{N_{i},R\right\}$ for *i* = 1, 2, ⋯ , *T*.
3. Take the majority vote according to the *T* predictions of {*F*_*i*_(*x*)|*i* = 1, 2, ⋯ , *T*}.

### 2.5. Evaluation Criteria

We used the following metrics to evaluate the performance of the classification models:

Accuracy: $\;ACC=\frac{TP+TN}{TP+FP+TN+FN}$

Recall: $\;REC=\frac{TP}{TP+FN}$

Specificity: $\;SPC=\frac{TN}{TN+FP}$

F_1_ score: $\;F_{1}=\frac{2TP}{2TP+FP+FN}$

Matthews correlation coefficient:

$$MCC=\frac{TP\times TN-FP\times FN}{\sqrt{\left(TP+FP\right)\left(TP+FN\right)\left(FP+TN\right)\left(FN+TN\right)}}$$

where

1. TP (true positive) is the number of cell lines labeled with sensitivity and predicted as sensitivity;
2. FP (false positive) is the number of cell lines labeled with resistance and predicted as sensitivity;
3. FN (false negative) is the number of cell lines labeled with sensitivity and predicted as non-sensivity;
4. TN (true negative) is the number of cell lines labeled with resistance and predicted as non-sensivity.

Besides the metrics above, AUC was also obtained.

Because the total number of samples was much smaller than that of the features, the above evaluation criteria were obtained by using 10-fold cross validation (CV). The dataset was randomly partitioned into 10 equal sized subsets. Of the ten subsets, a single subset was used as the test set to calculate the evaluation criteria of the models trained by the remaining nine subsets. The above process was then repeated 10 times, and the mean of the evaluation criteria obtained in the 10 times was used as the final criteria. In this way, the test datasets can be ensured to be independent of the training datasets.

## 3. Results

### 3.1. Data Description

There are missing data in both datasets. These missing data were compensated by using the weighted mean method described in the section Materials and Methods. The total numbers of samples for each variable are listed in Table 1.

**Table 1:** Total numbers of samples for three features.

**Dataset**	**State**	**Drugs**	**Cell lines**	***x*_*rna*_**	***x*_*snv*_**	***x*_*cna*_**
GDSC	Raw	139	1,124	11,833 (789)	70 (778)	24,960 (936)
	Preprocessed	98	555	11,712 (555)	54 (555)	24,959 (555)
CCLE	Raw	24	1,061	20,049 (1,028)	1,667 (1,044)	24,960 (742)
	Preprocessed	24	363	19,389 (363)	1,667 (363)	24,960 (363)

*The number in the parenthesis means a total of cell lines corresponding to the features*.

**Figure 2:**
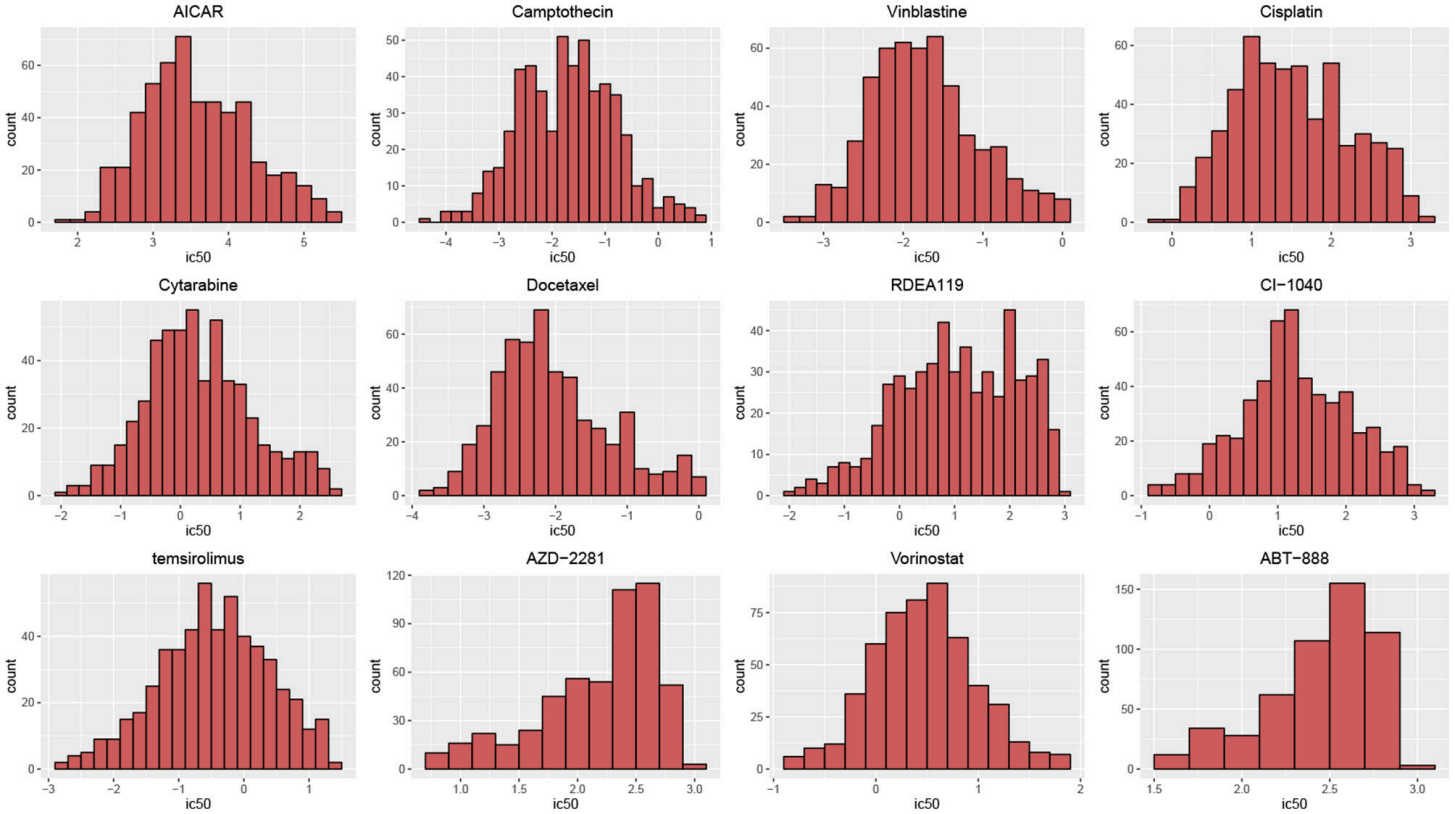
Histograms of drug responses for 12 drugs in GDSC. The distributions of drug responses were different for various drugs.

According to their histograms, the most of distributions of drug responses of cell lines in two datasets can be approximated by the Gauss distribution (Figure 2). *t*-hypothesis test showed that the significance of two groups divided by median of IC50 in GDSC is of *p*-values from 4.27 × 10^−160^ to 6.89 × 10^−46^; such significance in CCLE is of *p*-value from 7.14 × 10^−95^ to 4.05 × 10^−4^.

### 3.2. Prediction Performance of AutoBorutaRF

To illustrate the effectiveness of our AutoBorutaRF method, we demonstrated its prediction performance on GDSC and CCLE datasets. Meanwhile, we compared it with other four algorithms, including naive Bayes classifier (Barretina et al., 2012), SVM-RFE (Dong et al., 2015), FSelector for *k*-nearest-neighbors (KNN) algorithm (Soufan et al., 2015), and AutoHidden. The naive Bayes method first selected the top 30 features using either non-parametric Wilcoxon Sum Rank Test (for the gene expression features) or Fisher Exact Test (for the gene mutations). Then, the remaining significant features (*p* < 0.25) were clustered using a message-passing algorithm for each type of features. Then, they combined these two-part features and used a naive Bayes classifier for the drug response classification prediction. SVM-RFE is a wrapper method using a recursive feature selection and SVM classifier. The parameters of feature number, gamma and cost were set to be 10, 0.5, and 10, which were the optimal parameters selected by SVM-RFE. FSelector selected features using FSelector based on the information entropy and applied to the KNN algorithm. In AutoHidden, we directly use the hidden layer of the autoencoder constructed in our AutoBorutaRF, as the features.

**Figure 3:**
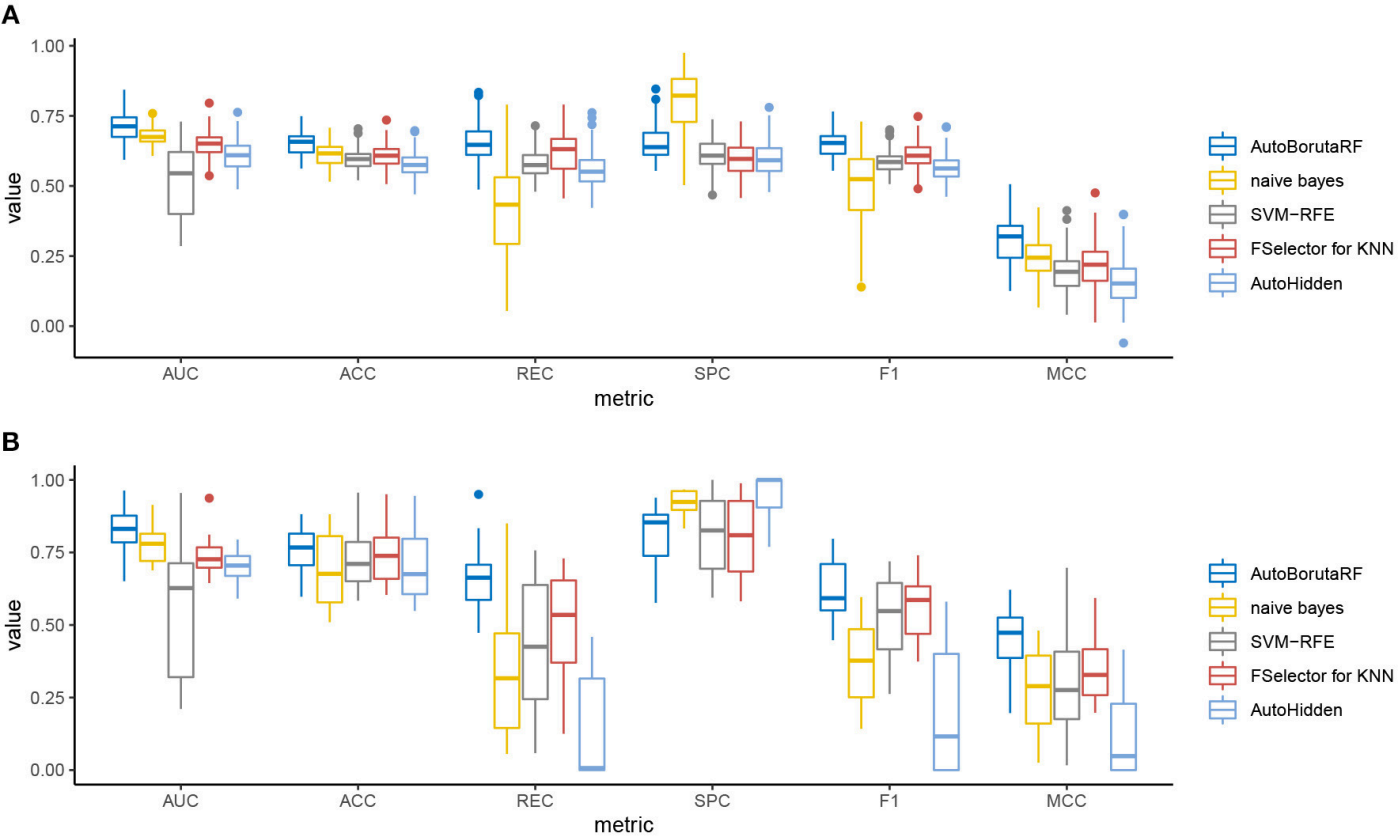
Box plots of the six evaluation metrics overall the cell lines in the **(A)** GDSC and **(B)** CCLE datasets. Our method was of the best performance with respect to AUC, accuracy, recall, specificity, *F*_1_ score, and Matthews correlation coefficient. The naive Bayes classifier and SVM-RFE outperformed at specificity.

The overall prediction performance of the five methods for the two datasets is illustrated in Tables 2, 3 and Figure 3. All the metrics in the figure were obtained by using 10-fold CV. Figure 3 showed that our method was of the best performance with respect to AUC, accuracy, recall, specificity, *F*_1_ score, and Matthews correlation coefficient.

**Table 2:** Mean values of six evaluation metrics obtained from GDSC.

**Method**	**AUC**	**ACC**	**REC**	**SPC**	***F*_1**	**MCC**
AutoBorutaRF	**0.7116**	**0.6534**	**0.6527**	0.6542	**0.6501**	**0.3109**
Naive Bayes	0.6792	0.6109	0.4242	**0.7969**	0.4947	0.2475
SVM-RFE	0.5159	0.5945	0.5797	0.6092	0.5855	0.1915
FSelector	0.6477	0.6061	0.6171	0.5952	0.6068	0.2155
AutoHidden	0.6095	0.5780	0.5576	0.5984	0.5651	0.1584

*The bold number indicates the best result*.

**Table 3:** Mean values of six evaluation metrics obtained from CCLE.

**Method**	**AUC**	**ACC**	**REC**	**SPC**	***F*_1**	**MCC**
AutoBorutaRF	**0.8210**	**0.7638**	**0.6560**	0.8137	**0.6248**	**0.4520**
Naive Bayes	0.7793	0.6838	0.3325	0.9194	0.3662	0.2759
SVM-RFE	0.5516	0.7287	0.4286	0.8129	0.5239	0.2961
FSelector	0.7372	0.7430	0.5061	0.8058	0.5639	0.3535
AutoHidden	0.7063	0.6970	0.1338	**0.9501**	0.3567	0.2198

*The bold number indicates the best result*.

Among the 98 drugs in GDSC, ABT-888 presented the worst prediction with AUC being 0.5935, and the best prediction is for RDEA119 with AUC being 0.8282. Meanwhile, RDEA119, PD-0325901, 17-AAG, and Vorinostat were the only four drugs with AUC >0.8. However, there were 59 drugs, whose AUCs were higher than 0.7. Among the 24 drugs in CCLE, the worst prediction is for AEW541 with AUC being 0.6509. The best three predictions are for Nutlin-3, LBW242, and AZD6244, with AUC being 0.9633, 0.9300, and 0.9079, respectively. The AUCs of Irinotecan, Panobinostat, PD-0332991, PD-0325901, PHA-665752, PLX4720, and Topotecan are higher than 0.85. The receiver operating characteristic (ROC) curves are listed in Supplementary File 1.

### 3.3. Identified Biomarkers Are Associated With Cancer and Drug Target Pathway

**Figure 4:**
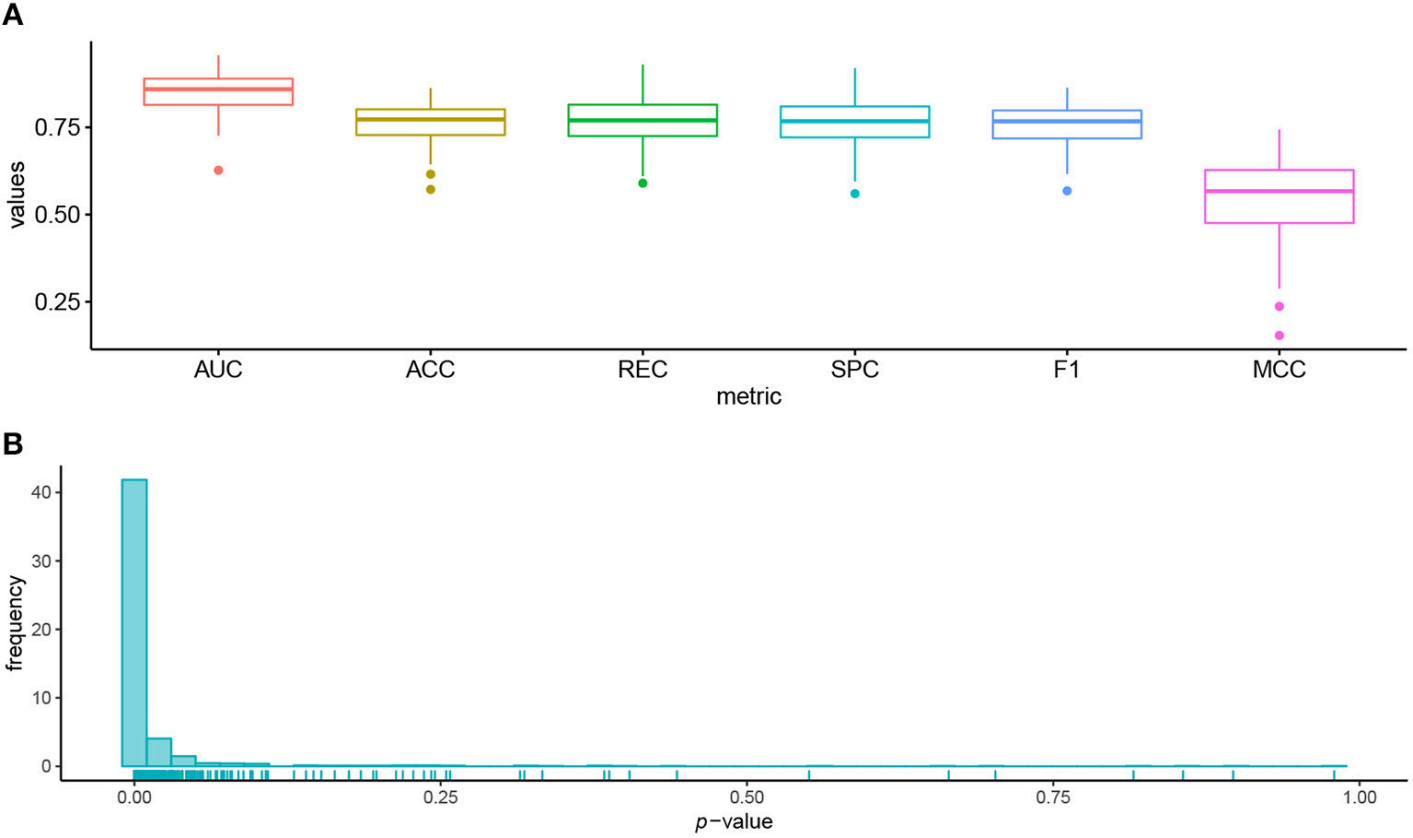
Prediction performance for the lung cell lines in GDSC. **(A)** Box plots of six metrics overall the lung cells showed the satisfying prediction performance. **(B)** Histogram of *p*-values obtained by the statistical significance test for the identified features proved that most of the identified features were of significantly different genetic profiles between the sensitive and non-sensitive populations.

We used 95 lung cell lines in the GDSC database to illustrate the biological significance of the identified biomarkers. Figure 4A shows the prediction performance of AutoBorutaRF for the lung cell lines. AutoBorutaRF showed satisfying prediction performance for predicting the drug responses for the lung cell lines. We used the non-parametric Wilcoxon sum rank test for the genetic features of gene expression and copy number alternation and a Fisher exact test for the genetic feature of single-nucleotide mutation, to test the significant difference of the genetic features between the sensitive and non-sensitive populations. Among all the identified 1,087 features (Supplementary File 2), a total of features with *p* < 0.05 was 1029, shown by Figure 4B. These results showed that most of the identified features were of significantly different genetic profiles between two classes (Supplementary File 3).

**Figure 5:**
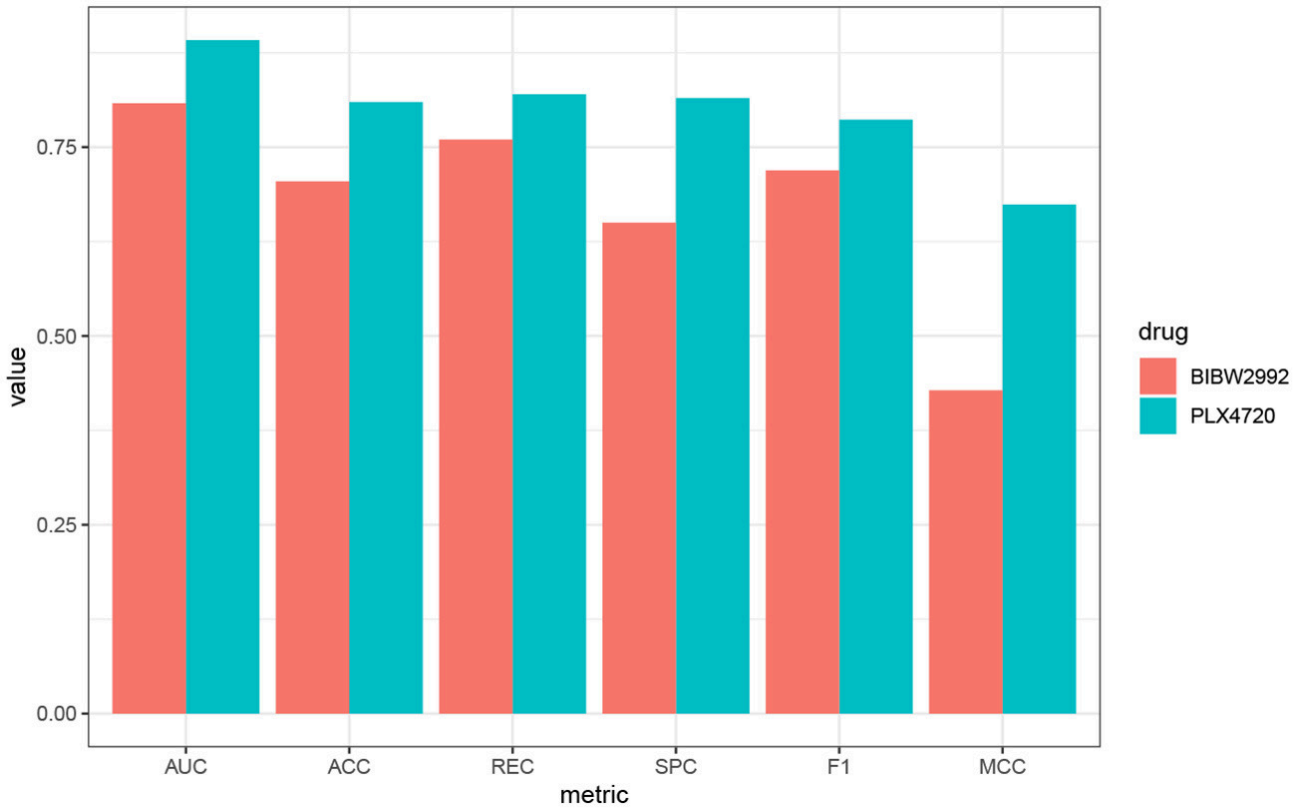
Performance metrics of AutoBorutaRF overall the lung cell lines in GDSC for PLX4720 and BIBW2992.

We further use PLX4720 and BIBW2992 as two examples to illustrate the biological significance of the features selected for the lung cell lines. Prediction metrics of these two drugs are shown in Figure 5. PLX4720 is the inhibitor for B-raf and targets at MAPK signaling pathway (Michaelis et al., 2014). The selected significant features for PLX4720 were *CCL19, CCRL2, CST7, GPR143, HDAC5*, and *IDO1*. *CCRL2* inhibits p38 MAPK phosphorylation and up-regulates the expression of E-cadherin (Wang et al., 2015). Besides, *CCR7, CST7, GPR143, HDAC5*, and *IDO1* are also related to lung cancer or the MAPK pathway (Liu et al., 2014, 2018; Li and Seto, 2016; Matthews et al., 2016;Rose et al., 2016).

BIBW2992 inhibits *ERBB2* and *EGFR* and targets at EGFR signaling pathway (Iorio et al., 2016) and has been widely investigated for cancers, like lung cancer and melanoma (Rinehart et al., 2004; Nehs et al., 2010; Varmeh et al., 2016). The selected significant features were *FYN, KCNH2, REST, CDH12, LRRC8E, SCG2, PHF8, PCSK1*, *ANXA2*, and *MIR6730*. *FYN* was an authentic Effector of oncogenic EGFR signaling, by limiting EGFR tumor cell motility (Lu et al., 2009). *CDH12* plays an important role in non-small-cell lung cancer(NSCLC) geneses, resulting from that the mutations of *CDH12* and other PRAME family members were equally distributed among tumors of different grades and stages (Bankovic et al., 2010). *SCG2* is in connection with the alteration of miRNA profiles in A549 human non-small-cell lung cancer cells (Shin et al., 2009). *KCNH2, REST, LRRC8E, PHF8, PCSK1, ANXA2, and MIR6730* have been also proved to be related to signaling pathway EGFR and lung cancer (Bonilla and Geha, 2006; de Castro et al., 2006; Kreisler et al., 2010; Wang et al., 2012; Demidyuk et al., 2013; Shen et al., 2014; D́ıaz-Rodŕıguez et al., 2018). The function descriptions and interaction networks of the identified features for PLX4720 and BIBW2992 are included in Supplementary File 4.

## Discussion

The prediction of anticancer drug response is crucial for many applications, like the preclinical setting and clinical trial design. The prediction models for drug response include regression models and classification models. This research developed AutoBorutaRF for predicting the drug response for a two-fold aim: achieving proper features for RF and investigating biologically significant biomarkers for the explaining drug response. Because the genetic feature candidates are a vast set, we cannot directly apply the well developed Boruta algorithm for feature selection. We first drastically reduced the dimension by constructing the autoencoder network. Different from the typical application of a hidden layer of the autoencoder, we extracted the inputs with large contributions evaluated by the Gedeon method.

Considering AUC = 0.7 as a pass mark, 22 of 24 drugs in CCLE were of qualified prediction performance; 59 of 98 drugs in GDSC were of qualified prediction performance. Further analysis should be conducted to investigate the reasons leading to the prediction difference between two datasets.

We further investigated the biological significance. We proved that most of the identified genetic features between the sensitive and non-sensitive cell lines were significantly different. By using PLX4720 and BIBW2992 as two examples, we illustrated that many genes identified by AutoBorutaRF were reported to have close relationship with tumorigenesis or cancer progression. The detailed function explanations and interaction networks of the selected features can be referred to Supplementary File 4. Thus, AutoBorutaRF can be considered to be a capable machine learning method for determining the biomarkers for predicting the drug response for the preclinical and clinical purposes.

Note that our proposed method used no prior information to obtain the optimal feature set in the sense of prediction performance. In future research, the pre-determined information, like pathway knowledge, and the prior distribution describing the uncertainties of anticancer drugs can be considered to be embedded in our method.

## Data Availability

The source code and datasets for this study can be downloaded from https://github.com/bioinformatics-xu/AutoBorutaRF.

## Author Contributions

XX and PQ processed the data, designed the algorithm, and the programming codes, and wrote the manuscript. YW supported result interpretation and manuscript writing. JW and HG supervised the project and contributed to writing the manuscript.

### Conflict of Interest Statement

The authors declare that the research was conducted in the absence of any commercial or financial relationships that could be construed as a potential conflict of interest.

## Supplementary Material

Supplementary File 1 ROC curve of ten-fold cross validation.

Supplementary File 2 Selected features.

Supplementary File 3 Results of feature significance test.

Supplementary File 4 Function descriptions and interaction networks for PLX4720 and BIBW2992.
