# Supplementary material for: Autoencoder Based Feature Selection Method for Classification of Anticancer Drug Response
Source: Front Genet. 2019 Mar 27;10:233. doi: 10.3389/fgene.2019.00233 (PMC6445890; doi:10.3389/fgene.2019.00233)
Supplement: Supplementary File 1 — ROC curve of ten-fold cross validation. [file Data_Sheet_1.zip › supplementary20180113/Supplementary File 1--ROC curve of ten-fold cross validation/Supplementary File 1--ROC of GDSClung.pdf]

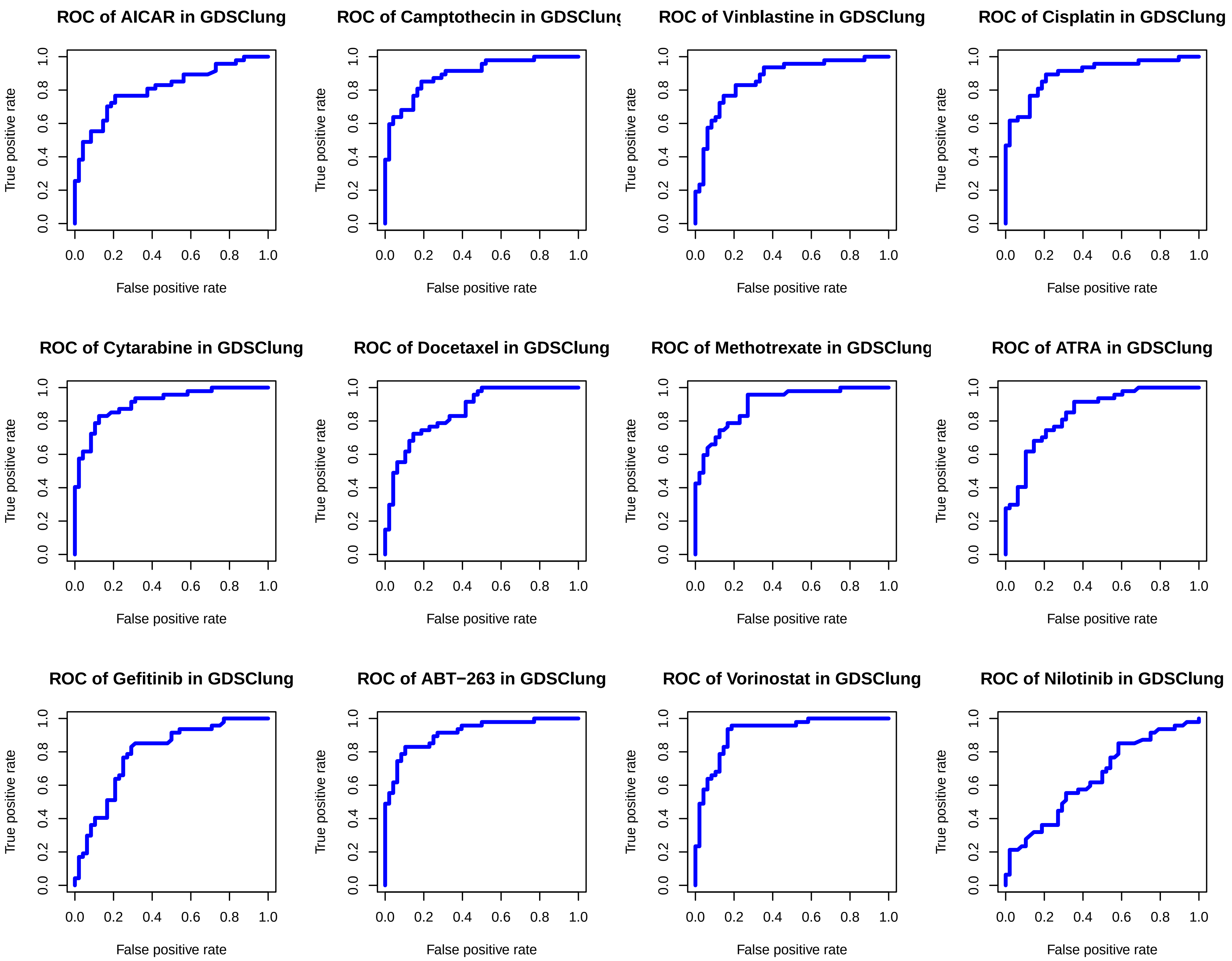
ROC of AICAR in GDSClung
ROC of Camptothecin in GDSClung
ROC of Vinblastine in GDSClung
ROC of Cisplatin in GDSClung
ROC of Cytarabine in GDSClung
ROC of Docetaxel in GDSClung
ROC of Methotrexate in GDSClung
ROC of ATRA in GDSClung
ROC of Gefitinib in GDSClung
ROC of ABT−263 in GDSClung
ROC of Vorinostat in GDSClung
ROC of Nilotinib in GDSClung
True positive rate
False positive rate
0.0
0.2
0.4
0.6
0.8
1.0
