# Supplementary material for: Autoencoder Based Feature Selection Method for Classification of Anticancer Drug Response
Source: Front Genet. 2019 Mar 27;10:233. doi: 10.3389/fgene.2019.00233 (PMC6445890; doi:10.3389/fgene.2019.00233)
Supplement: Supplementary File 1 — ROC curve of ten-fold cross validation. [file Data_Sheet_1.zip › supplementary20180113/Supplementary File 1--ROC curve of ten-fold cross validation/Supplementary File 1--ROC of GDSC.pdf]

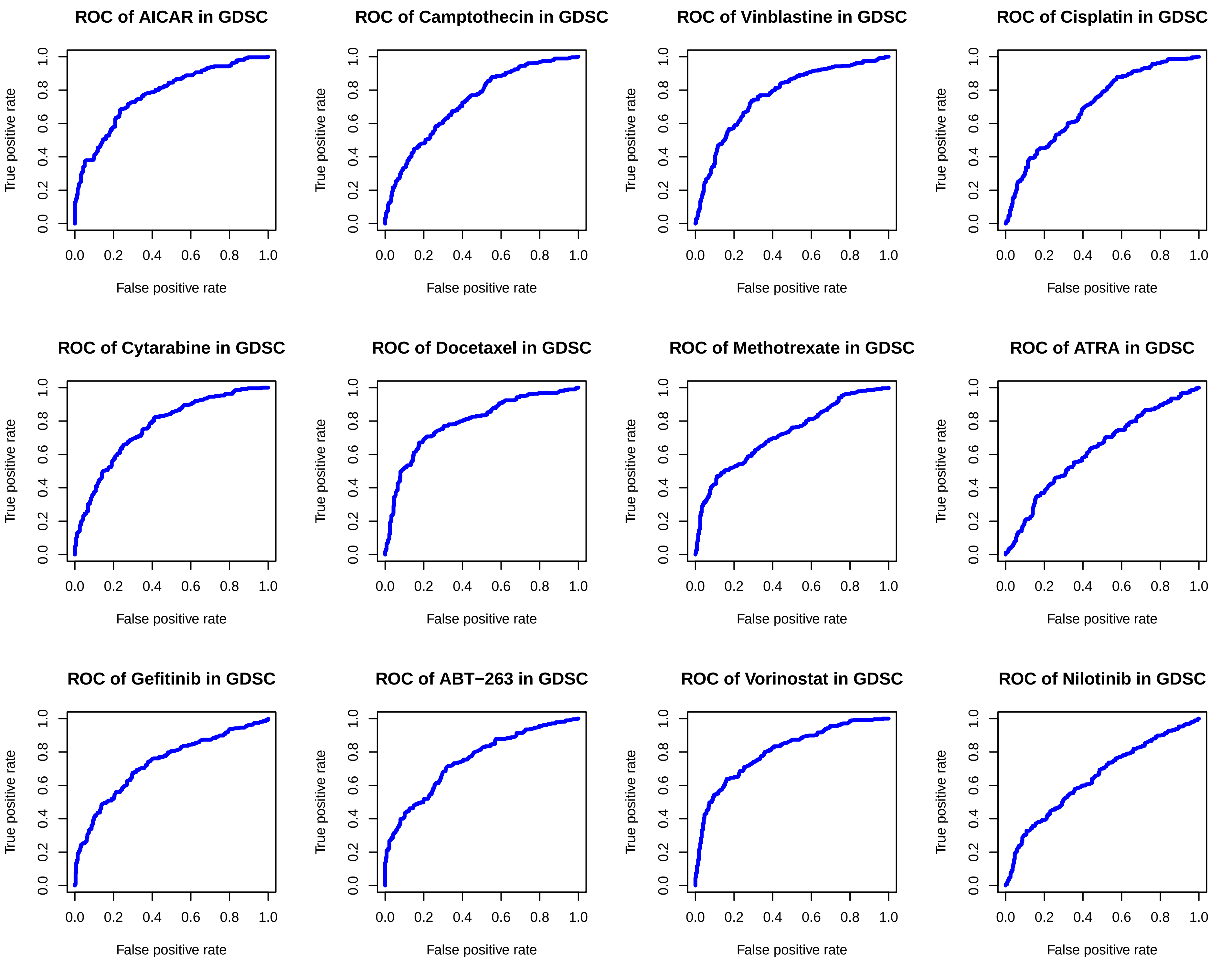

ROC of AICAR in GDSC
ROC of Camptothecin in GDSC
ROC of Vinblastine in GDSC
ROC of Cisplatin in GDSC
ROC of Cytarabine in GDSC
ROC of Docetaxel in GDSC
ROC of Methotrexate in GDSC
ROC of ATRA in GDSC
ROC of Gefitinib in GDSC
ROC of ABT−263 in GDSC
ROC of Vorinostat in GDSC
ROC of Nilotinib in GDSC
True positive rate
False positive rate
0.0
0.2
0.4
0.6
0.8
1.0
