# Supplementary material for: Autoencoder Based Feature Selection Method for Classification of Anticancer Drug Response
Source: Front Genet. 2019 Mar 27;10:233. doi: 10.3389/fgene.2019.00233 (PMC6445890; doi:10.3389/fgene.2019.00233)
Supplement: Supplementary File 1 — ROC curve of ten-fold cross validation. [file Data_Sheet_1.zip › supplementary20180113/Supplementary File 1--ROC curve of ten-fold cross validation/Supplementary File 1--ROC of CCLE.pdf]

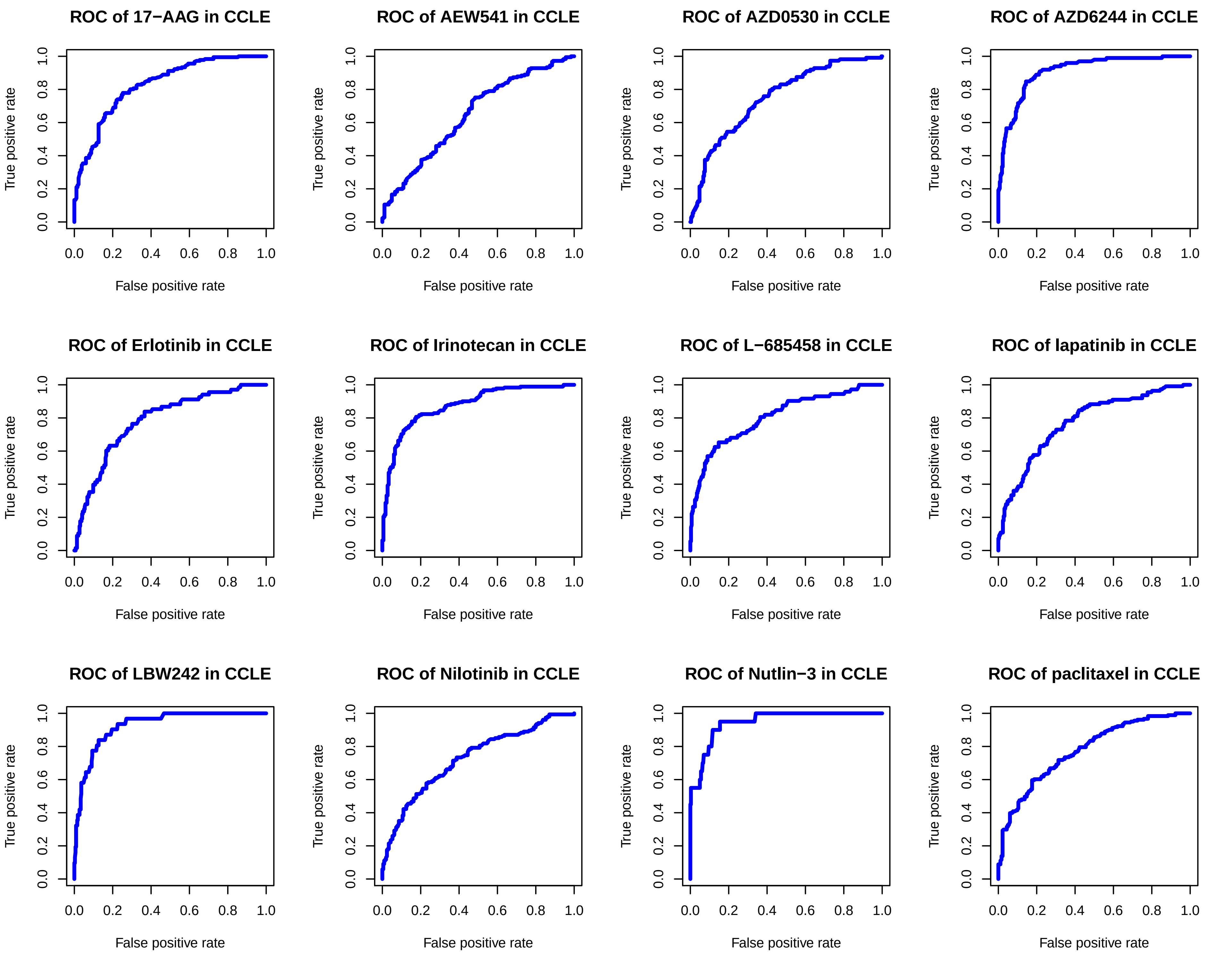
ROC of 17−AAG in CCLE
ROC of AEW541 in CCLE
ROC of AZD0530 in CCLE
ROC of AZD6244 in CCLE
ROC of Erlotinib in CCLE
ROC of Irinotecan in CCLE
ROC of L−685458 in CCLE
ROC of lapatinib in CCLE
ROC of LBW242 in CCLE
ROC of Nilotinib in CCLE
ROC of Nutlin−3 in CCLE
ROC of paclitaxel in CCLE
True positive rate
False positive rate
0.0
0.2
0.4
0.6
0.8
1.0
