# Supplementary material for: Autoencoder Based Feature Selection Method for Classification of Anticancer Drug Response
Source: Front Genet. 2019 Mar 27;10:233. doi: 10.3389/fgene.2019.00233 (PMC6445890; doi:10.3389/fgene.2019.00233)

**Function descriptions and** **interaction networks of selected genes for** **PLX4720 and BIBW2992**

1. Functions descriptions

(A)

**PLX4720**

| ***Impor genes*** | ***Description*** |
| --- | --- |
| ***CCL19*** | CCL19 is reported as a strong activator to MAPK on a molar basis, who first induce the CCR7 pathway and the MAPK members are the downstream molecules of the CCR7 pathway. The study carried out by Hung-Wei Cheng et al. suggest that a distinct population of CCL19-producing FSCs fosters the development of an immune-stimulating intratumoral niche for immune cells to control cancer growth. |
| ***CCRL2*** | A heptahelic transmembrane receptor that shows the highest degree of homology with CCR1, an inflammatory chemokine receptor. It inhibits p38 MAPK phosphorylation and up-regulates the expression of E-cadherin. The research of Karel Otero et al propose that CCRL2 is a new potential target for therapeutic strategies aimed at controlling lung hypersensitivity. |
| ***CST7*** | It has been shown that CST7 contains a unique C/EBP α binding site that might account for restricted expression in immune cells. CST7 expresses at high levels in the lung microenvironments, which is a reliable trigger of lung cancers. |
| ***GPR143*** | GPR143 shows high constitutive activity in the b-arrestin assay, was inhibited by several compounds. GPR143 is also expressed in the central nervous system and some peripheral tissues (kidney, spleen, and lung). |
| ***HDAC5*** | According to a study presented by Yixuan Li et al, the low expression of HDAC5 is associated with poor prognosis in lung cancer patients. Fan et al. proved that the knockdown of HDAC5 leads to a significant up-regulation of p21 and down-regulation of cyclin D1 and CDK2/4/6, which results in G1- phase cell-cycle arrest in human HCC cells. |
| ***IDO1*** | IDO is a nodal pathogenic driver of lung cancer and metastasis development. The study carried out by Cancer Discov et al. defines IDO as a prototypical integrative modifier that bridges inflammation, vascularization and immune escape to license primary and metastatic tumor outgrowth. |

(B)

**BIBW2992**

| ***Impor genes*** | ***Description*** |
| --- | --- |
| ***FYN*** | FYN is a strong effector of oncogenic EGFR signaling, enhancing invasion and tumor cell survival in vivo. EGFR signaling activates FYN to enhance glioblastoma invasion and tumor survival in vivo. A study carried out by Kan V. Lu shows that FYN inhibition by either genetic or pharmacologic means greatly limits tumor invasion and promotes tumor cell apoptosis. |
| ***PHF8*** | PHF8 is an oncogenic protein in human non-small cell lung cancer. PHF8 regulates lung cancer cell proliferation and cellular transformation. Yuzhou Shen et al. found that PHF8 knockdown induces DNA damage and apoptosis in lung cancer cells. PHF8 promotes miR-21 expression in human lung cancer, and miR-21 knockdown blocks the effects of PHF8 on proliferation and apoptosis of lung cancer cells. It promotes lung cancer cell growth and survival by regulating miR-21. |
| ***PCSK1*** | PCSK1 expression is largely detected in cancers with neuroendocrine features, in particular, SCLC. The changes in the expression of PCSK1 gene in lung cancer have a limited number of scenarios, which may correspond to previously undetected NSCLC types, according to the study presented by Ilya V. Demidyuk et al. |
| ***CDH12*** | It is revealed that CDH12 plays important role in non-small-cell lung cancer(NSCLC) geneses, resulting from that the mutations of CDH12 and other PRAME family members were equally distributed among tumors of different grades and stages. |
| ***ANXA2*** | According to Chi-Yun Wang et al, ANXA2 is highly expressed in non-small cell lung cancer (NSCLC) and is positively correlated with a poor prognosis. NSCLC A549 cells lacking ANXA2 exhibited defects in tumor growth in vivo and in cell proliferation in vitro without cytotoxicity. |
| ***SCG2*** | SCG2 is in connection with the alteration of miRNA profiles in A549 human non-small-cell lung cancer cells. |

2. Interaction networks of selected genes

(A) PLX4720


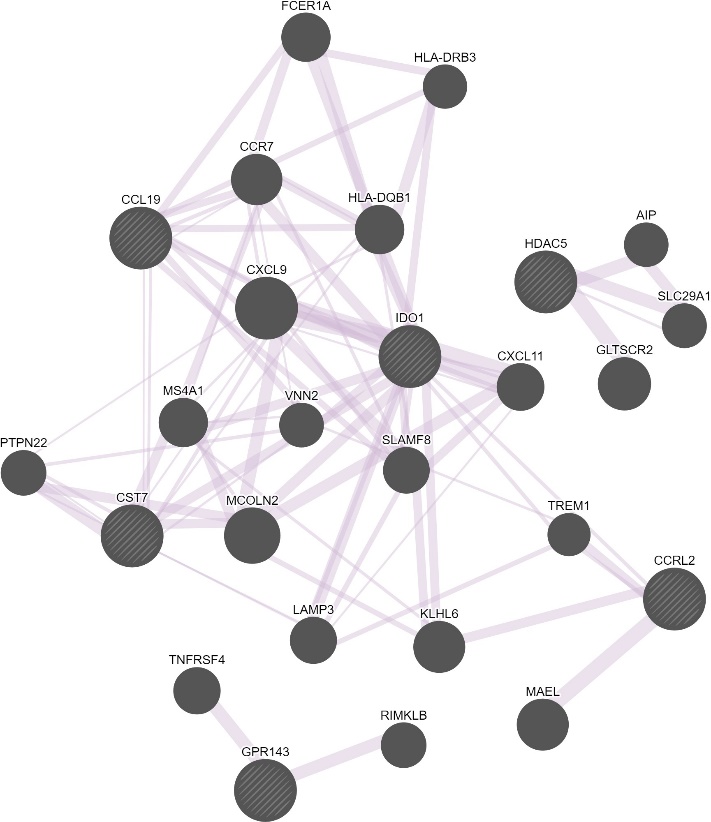


(B) BIBW2992


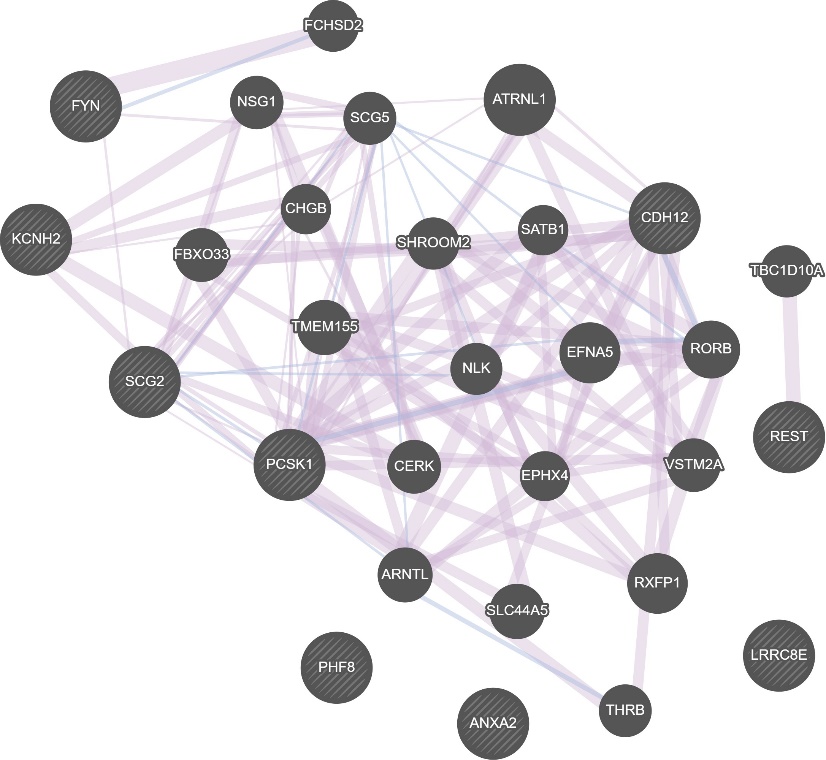

Supplement: Supplementary File 1 — ROC curve of ten-fold cross validation. [file Data_Sheet_1.zip › supplementary20180113/Supplementary File 4-- Function_descriptions_and_interaction_networks.docx]
