# Supplementary figures and images for: Autoencoder Based Feature Selection Method for Classification of Anticancer Drug Response
Source: Front Genet. 2019 Mar 27;10:233. doi: 10.3389/fgene.2019.00233 (PMC6445890; doi:10.3389/fgene.2019.00233)

# GDSC features significance test

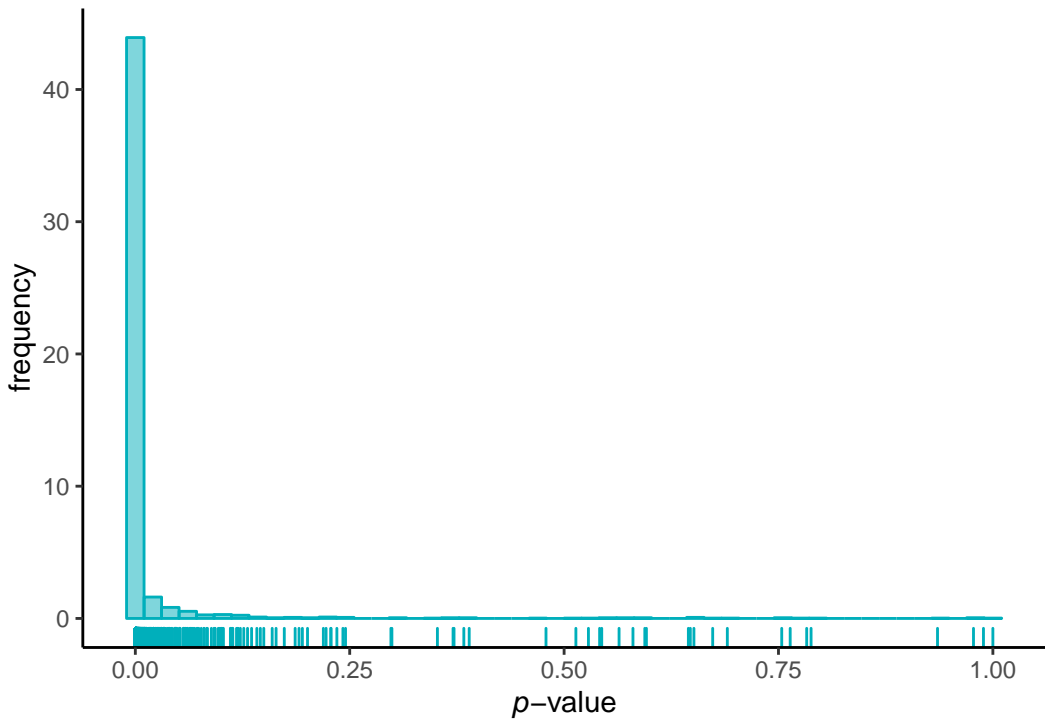

Supplement: Supplementary File 1 — ROC curve of ten-fold cross validation. [file Data_Sheet_1.zip › supplementary20180113/Supplementary File 3--Feature significance test/Supplementary File 3--histgram of GDSC features significance test.pdf]

CCLE features significance test

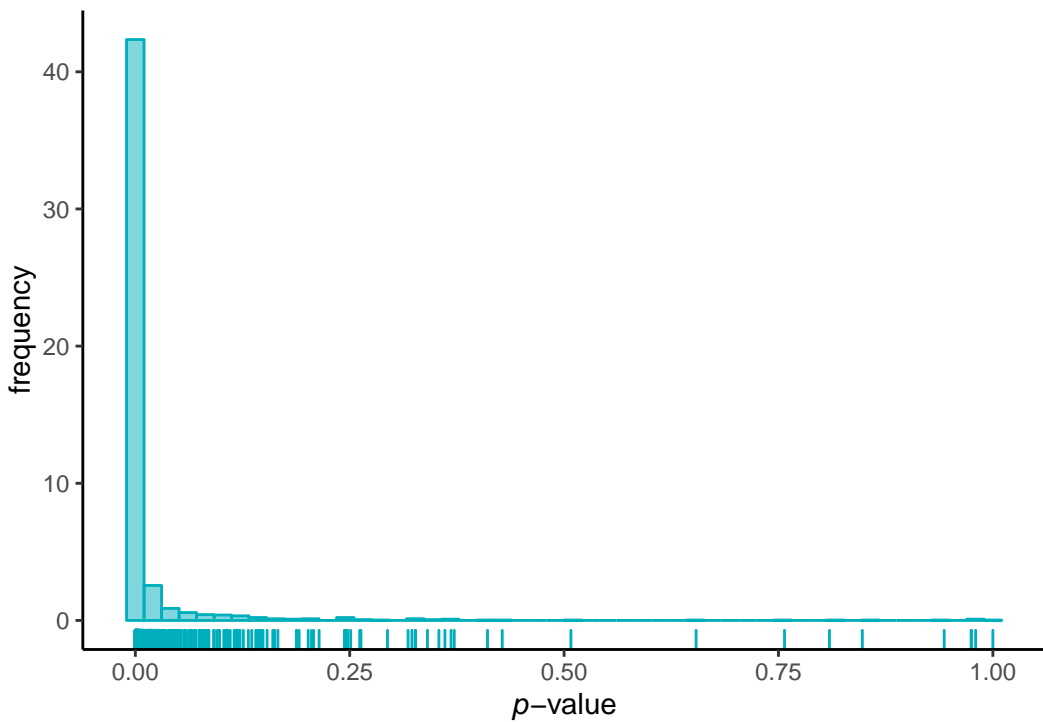

Supplement: Supplementary File 1 — ROC curve of ten-fold cross validation. [file Data_Sheet_1.zip › supplementary20180113/Supplementary File 3--Feature significance test/Supplementary File 3--histgram of CCLE features significant test.pdf]

# GDSClung features significance test

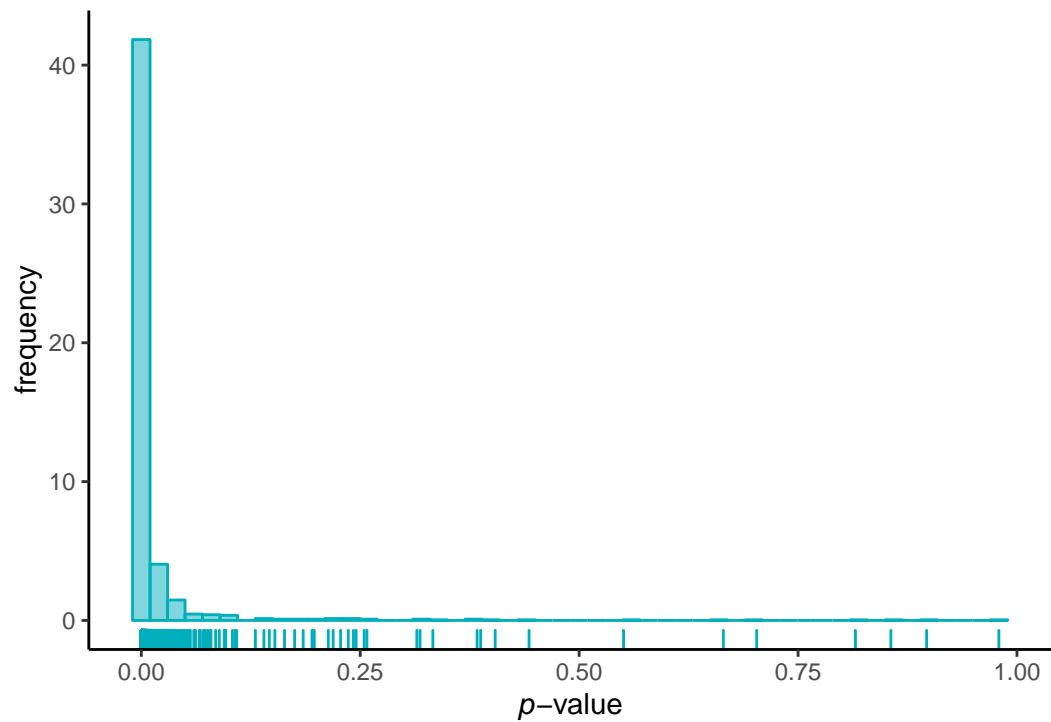

Supplement: Supplementary File 1 — ROC curve of ten-fold cross validation. [file Data_Sheet_1.zip › supplementary20180113/Supplementary File 3--Feature significance test/Supplementary File 3--histgram of GDSClung features significance test.pdf]
